# Synergistic Effects of Graphene and Ammonium Polyphosphate Modified with Vinyltrimethoxysilane on the Properties of High-Impact Polystyrene Composites

**DOI:** 10.3390/polym13060881

**Published:** 2021-03-13

**Authors:** Xianghui Shi, Yong Pan, Yuguo Wang, Zhimeng Jia, Tingting Chen, Junhui Gong, Juncheng Jiang

**Affiliations:** 1 College of Safety Science and Engineering, Nanjing Tech University, Nanjing 211816, China; shixianghui@foxmail.com (X.S.); 13934911784@163.com (Y.W.); ars-zjia@njtech.edu.cn (Z.J.); ctt3187@njtech.edu.cn (T.C.); gjh9896@njtech.edu.cn (J.G.); ypnjut@126.com (J.J.); 2 School of Environment & Safety Engineering, Changzhou University, Changzhou 213164, China

**Keywords:** ammonium polyphosphate, graphene, high-impact polystyrene, thermal stability, flame retardancy

## Abstract

Ammonium polyphosphate (APP) was modified with a silane coupling agent (vinyltrimethoxysilane, Si-171), and then the synergistic flame retarding effect of graphene and surface-modified APP (APP@Si-171) on high-impact polystyrene (HIPS) was investigated. Surface modification and thermal stability characterization of APP were analyzed by Fourier transform infrared spectroscopy (FTIR), energy dispersive spectrometer (EDS), scanning electron microscopy (SEM) and thermogravimetric analysis (TGA). The results showed that surface-modified APP (APP@Si-171) exhibited significantly better dispersion and less agglomeration tendencies compared with pure APP. A series of target HIPS composites containing different mass fractions of the two flame retardants were prepared by melt blending. TGA and cone calorimeter tests (CCT) were conducted to quantitatively investigate the thermal and flammability properties of the composites, respectively. Results from TGA and CCT demonstrated that the addition of the flame retardants delayed the onset and peak temperatures in differential thermogravimetry (DTG) curves and weakened the peak heat release rate (PHRR) and total heat release (THR). Moreover, the synergistic effect index (SE) was employed to quantify the synergistic behavior between the two fillers, and the results showed that APP@Si-171 and graphene had a synergistic effect on improving the thermal stability and flame retardancy of HIPS.

## 1. Introduction

High impact polystyrene (HIPS) is a widely used thermoplastic copolymer in both engineering and household applications, which is synthesized by grafting a small amount of elastomer polybutadiene into the polystyrene matrix, due to its excellent rigidity, ease of coloring and processing, good dimensional stability, high impact strength, etc. [1]. However, the high flammability and smoke production during burning limit its application [2,3]. According to the data of the Fire Rescue Bureau of the Ministry of Emergency Management of the People’s Republic of China, in 2018, electrical fires accounted for 34.6% of the total number of fires in the whole year, ranking first in all kinds of fires. To reduce the fire risk, some methods were developed, such as fire-retardant coating technology, flame-retardant filler technology, flame-retardant solution immersion and chemical grafting flame-retardant technology [4,5,6]. Among them, flame-retardant filler technology had become one of the most commonly used technologies due to its low cost and excellent performance.

Ammonium polyphosphate (APP) is one of the most common flame retardants because it has the advantages of environmental protection, low cost and good flame retardancy. However, since the shortcomings, such as water solubility and poor compatibility in polymers, limit the application of APP, it is necessary to improve the compatibility of APP to meet the corresponding requirements. Currently, treating APP with a coupling agent is an effective way to solve the shortcomings [7,8]. In addition, APP can only be used as an acid source and not as a carbon source, since it does not contain carbon element [9,10]. Therefore, APP needs to be applied together with fillers that can be used as a carbon source to obtain better flame retardancy [11,12].

Graphene is a kind of two-dimensional material with a thickness of only one carbon atom, which consists of carbon atoms and SP^2^ hybrid orbital [13]. Graphene shows excellent optical, electrical and mechanical properties, leading to promising applications in materials, micro-nano processing, energy, biomedicine and drug delivery [14,15]. Furthermore, graphene can not only be used as a flame retardant, but also enhance the thermal stability and mechanical properties of materials [16]. Therefore, graphene is a very suitable carbon source.

Recently, to further improve the flame retardancy, two or more flame retardants had been added simultaneously into the polymer matrix to achieve the optimization purpose. Amani et al. [17] studied the effect of mixing condition on the mechanical and thermal properties of polyethylene/high impact polystyrene/graphite oxide (PE/HIPS/GO) polymer nanocomposites. When 0.6 wt% GO was added, the tensile modulus of the composite increased by 12% and the temperature for 10% weight loss increased by about 25 °C, which indicated that GO could be used as a reinforcing filler in polymer blends, thus significantly improving its mechanical property and thermal stability. Rostampour et al. [18] revealed that the co-existence of GO and clay had synergistic effect on the tensile modulus and flexural strength of ternary composites. The viscoelastic test results and TGA and DMTA test results also showed that the thermal stability and storage modulus of GO-clay modified HIPS matrix were enhanced. Song et al. [19] found that the addition of CNT and reduced graphene oxide (RGO) significantly reduced the peak heat release rate (PHRR), the average mass loss rate (AMLR) and the relative oxygen permeability coefficient of the PP composites, increased the storage modulus and showed significant synergistic effect on viscoelasticity, gas barrier and flame retardancy. Han et al. [20] found that GO or graphene could promote the carbonization of unburned fillers on the polymer surface, and the high heat resistance of GO and graphene could also promote the carbonization, which was helpful to improve the flame retardancy of polystyrene. Jiao et al. [9] used ethanolamine (ETA) to chemically modify APP, and the prepared new flame retardant, APP@ETA, was applied to thermoplastic polyurethane (TPU). APP@ETA could form a flame-retardant char layer and then inhibited the heat release and smoke generation in the early stage of the combustion of materials, which prevented further combustion of internal flammable materials and thus achieved good smoke suppression and flame-retardant effect. Gao et al. [21] used polydimethyl siloxane (PDMS) to optimize the traditional sol-gel method to improve the properties of silica-gel microencapsulated ammonium polyphosphate (SiOAPP), and applied them in TPU. The results showed that the mechanical properties and flame retardancy of TPU were greatly improved. Guo et al. [22] used the expandable graphite (EG) and ammonium polyphosphate modified with 3-(methylacryloxyl) propyltrimethoxy silane (M-APP) to improve the flame retardancy of the wood-polypropylene composites (WPC). The results showed that EG and M-APP can effectively improve the flame retardancy of WPC, and with the increase of EG content, the effect became stronger. When the ratio of M-APP and EG additives was 1:1, the LOI value reached the best, and the thermal degradation stability of WPC composites was greatly improved. Meng et al. [23] studied the effect of EG and APP on the flame retardancy and mechanical properties of rigid polyurethane foam (RPUF). The results indicated that both EG and APP could effectively interdict the burning of RPUF. It led to the increase of high temperature residue, which acted as a protective thermal barrier in the flame retardant process of RPUF. In addition, the mechanical properties of RPUF were improved after the addition of EG and APP. Zhu et al. [24] studied the synergistic effect of EG and APP on flame retardant polylactic acid (PLA). It was found that optimal synergism occurred in PLA/APP/EG system when the mass ratio of PLA/APP/EG was 1:3. The decomposed products of APP/PLA filled the gaps between EG sheets and formed a stable and dense carbon protective layer. This may be the reason for the synergistic effect of APP and EG in the PLA matrix. Tang et al. [25] revealed that there were synergistic effects of APP and red phosphorus masterbatch (RPM) with EG in the flame-retardant high-density polyethylene/ethylene vinyl-acetate copolymer (HDPE/EVA) composites. The compact and stable charred residues promoted by APP and RPM with EG prevented further combustion of the underlying polymer materials. The above studies showed that the synergistic effect between flame-retardant fillers was usually determined by comparing the experimental values of various properties of composite materials. However, the improvement of composite properties due to the synergistic effect between fillers had not been quantitatively determined. Therefore, the synergistic effect index (SE) should be employed to quantify the synergistic behavior of the surface-modified APP and graphene. In addition, to the best of our knowledge, no studies have been reported about the synergistic effects between surface-modified APP and graphene on the flame retardancy of HIPS matrix.

In this paper, silane coupling agent Si-171 (vinyltrimethoxysilane) was firstly used to surface modify APP to produce APP@Si-171, and it was added to HIPS in different proportions with graphene. A series of HIPS/APP@Si-171/graphene composites were prepared by melting blending method. The dispersion of the fillers was investigated by scanning electron microscope (SEM), and the thermogravimetric analysis and cone calorimeter tests were conducted to examine the synergetic effect of APP@Si-171 and graphene on thermal and flammability properties of the HIPS composites.

## 2. Experimental

### 2.1. Materials

HIPS-622 (MFI = 4.8 g/10 min; 200 °C, 5 kg) pellets were purchased from Shanghai SECCO Petrochemical Co. Ltd. (Shanghai, China). Multilayer graphene was obtained from Suzhou Tanfeng Graphene Technology Co. Ltd. (Suzhou, China). APP (phase II, the degree of polymerization >1000, APP content 99.75 mass%) was purchased from Hangzhou JLS Flame Retardants Chemical Co., Ltd. (Hangzhou, China). Silane coupling agent Si-171 (vinyltrimethoxysilane, Si-171 content 98.0 mass%), purchased from Nanjing Chemical Reagent Co. Ltd. (Nanjing, China), was used to modify APP. Analytical grade ethanol was purchased from Wuxi Yasheng Chemical Co. Ltd. (Jiangsu, China).

### 2.2. Surface Modification of Ammonium Polyphosphate

To modify APP, 1.5 g Si-171 liquid and 50 mL anhydrous ethanol were added into a 100 mL beaker and mixed by a magnetic stirrer for about 10 min until they were evenly mixed. Then, the solution was transferred into a 500-mL three-port flask equipped with a reflux condensing device, and the temperature was raised to 85 °C and maintained for 1 h under the stirring condition. Fifty g APP was dispersed in 200 mL anhydrous ethanol, which was fully stirred by an electric mixer for about 30 min to achieve uniformity. Then, the anhydrous ethanol solution of APP was added into the three-port flask with a reflux condensation device, and the reaction system temperature was kept at 50 °C for 2 h under stirring condition until the reaction was complete. Finally, the product was filtered and dried in a vacuum drying oven at 80 °C for 4 h. The dried product was grinded, crushed and filtered with a 300-mesh screen to obtain the modified product as fine powder.

### 2.3. Preparation of Composites

The melt blending method was employed to prepare the target HIPS/APP@Si-171/graphene composites. HIPS pellets were blended with APP, APP@Si-171 and graphene (HIPS+APP, HIPS+APP@Si-171, HIPS+graphene and HIPS+APP@Si-171+graphene), respectively, at 180 °C and 50 rpm for 20 min using a torque rheometer (CTR-300, Shanghai Changkai M&E Technology Co., Ltd., Shanghai, China). After the blending, the composites were pressed at 190 °C under the pressure of 10 MPa for 10 min to obtain 4 mm sheets, which were subsequently cut into a suitable size for further analysis. The mixing weight fractions of HIPS and fillers are given in Table 1.

### 2.4. Measurements and Characterization

Fourier transform infrared spectroscopy (FTIR) was performed with a Nicolet iS50 spectrometer (Thermo Fisher, Waltham, MA, USA). The range of test wavenumber was 4000–400 cm^−1^ and the resolution was 4 cm^−1^.

The energy dispersive spectrometer (EDS) tests were conducted with a tabletop microscope (TM3000, HITACHI, Tokyo, Japan) and energy dispersive spectrometer (X-act, Oxford, UK).

The composites were sputter-coated with gold and then the dispersion of the composites was examined with EVO-18 SEM (Zeiss, Germany).

The static contact angle tests were conducted on a contact angle equipment (DSA100, KRUSS, Germany) based on the sessile drop method, i.e., depositing a drop of test liquid onto the sample surface. The contact angle values of the samples in this study were the average values of at least five repetitive tests on the same sample. The average and standard deviation of the contact angle values were calculated as less than ±2.

The cone calorimeter tests (CCT) were conducted with a CC-2 cone calorimeter (Govmark Testing Services, Inc., USA) to investigate the flammability of HIPS and its composites according to ISO5660 (Reaction-to-fire Tests-Heat release, smoke production and mass loss rate). The samples, 100 × 100 × 4 mm^2^ in size, were horizontally exposed to the conic heat radiator with an external heat flux of 50 kW/m^2^. Each test was replicated at least three times to guarantee the reproducibility.

Thermogravimetric analysis (TGA) tests were performed with a 3+1100LF TGA sync analyzer (Mettler Toledo, Switzerland) operating in nitrogen atmosphere. Each test, containing 6–8 mg of sample, was carried out in a 70 μL alumina crucibles from 25 to 1000 °C at a heating rate of 10 °C/min under a purge gas flow of 40 mL/min. Additionally, each test was repeated at least three times.

### 2.5. Quantifying the Synergistic Effect in HIPS

Dittrich et al. [26] defined the synergistic effect as those two additives produced a greater effect when combined than the sum of the two additives when used alone. In this paper, Equation (1) [27] is employed to calculate the synergistic effect index (SE) and evaluate the synergistic behavior. Taking the calculation of the SE of PHRR as an example, the calculation can be written in the form of Equation (1):

(1) $$SE_{PHRR}=\frac{PHRR_{H+xAPP+yG}-PHRR_{H}}{\frac{x}{x+y}\left(PHRR_{H+\left(x+y\right)APP}-PHRR_{H}\right)+\frac{y}{x+y}\left(PHRR_{H+\left(x+y\right)G}-PHRR_{H}\right)}$$

where *x* and *y* are the mass fractions of the two additives, respectively, *H* is HIPS and *G* is grarphene. When the *SE* value is greater than 1, the two additives show the synergistic effect; when the *SE* value is equal to 1, the two additives show the superposition effect; when the *SE* value is less than 1, the two additives show the antagonistic effect.

## 3. Results and Discussion

### 3.1. Surface Modification and Thermal Stability Characterization of APP

**Figure 1:**
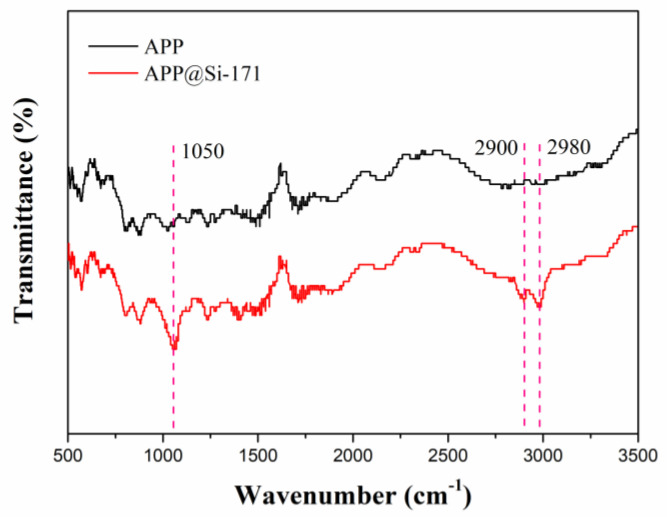
FTIR diagram of APP and APP@Si-171.

FTIR was used to explore the surface modification of APP. As shown in Figure 1, compared with APP, the spectra of APP@Si-171 had absorption peaks at near 1050 cm^−1^, 2900 cm^−1^ and 2980 cm^−1^. The peaks at 2900 cm^−1^ and 2980 cm^−1^ corresponded to the stretching vibration of C-H on the -Si-CH- group, and the peak at 1050 cm^−1^ was attributed to the stretching vibration of Si-O [28]. FTIR teats indicated that there are Si-containing groups in APP@Si-171, which was a proof of successful modification.

**Figure 2:**
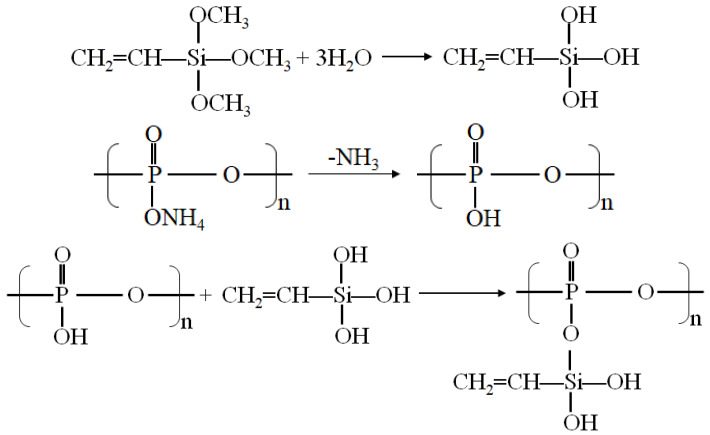
Reaction mechanism of APP modified with Si-171.

In combination with the results of the FTIR test, the reactions that may occur during the modification process are listed in Figure 2. -ONH_4_ in APP molecule loses NH_3_ to form -OH under heating condition [29], -OCH_3_ in Si-171 molecule hydrolyzes to form -OH, and both Si-OH and P-OH groups are dehydrated and condensed to form the Si-O-P structure, which connects the Si-171 molecule to the APP molecule. CH_2_=CH- in the Si-171 molecule enhances the dispersion and compatibility of APP@Si-171 in HIPS, and improves the moisture absorption of the APP molecule.

**Figure 3:**
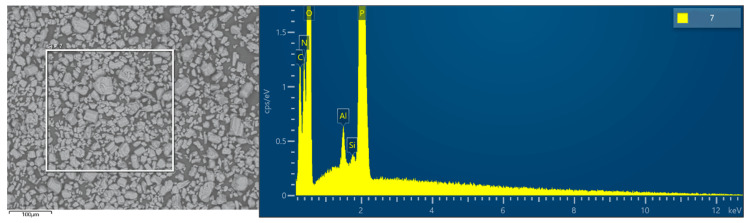
Energy dispersive spectrometer (EDS) analysis of APP@Si-171.

As can be seen from Figure 3, there is an obvious Si peak in APP@Si-171 spectrum, while there is no Si in APP, indicating the presence of Si-containing groups in APP@Si-171. Moreover, we can also infer that the distribution of Si in APP@Si-171 sample is relatively uniform.

According to the test analysis of the electron energy spectrum weight and atomic percentage of APP@Si-171 in Table 2, new C and Si appear on the surface of APP@Si-171, further proving that Si-171 has made obvious surface modification of APP, resulting in significant changes for surface structure of the modified APP.

**Figure 4:**
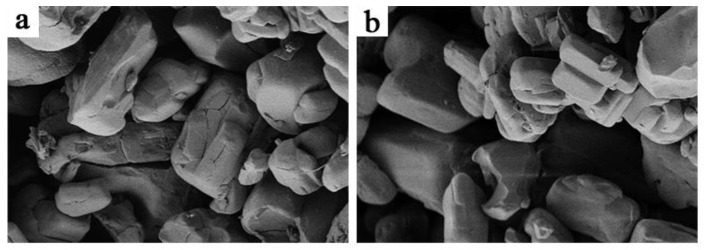
SEM images of APP (**a**) and APP@Si-171 (**b**) (×2000).

Figure 4 illustrates the micromorphology of the APP and APP@Si-171. It can be found that for the unmodified APP sample, the particle surface is rough and uneven, indicating higher hygroscopicity of the pure APP. After modification, the particle surface gets smoother and mellower, which infers that the APP particles are coated with membrane formed by Si-171 and the hydrophobicity of the surfaces of APP particles is improved significantly.

**Figure 5:**
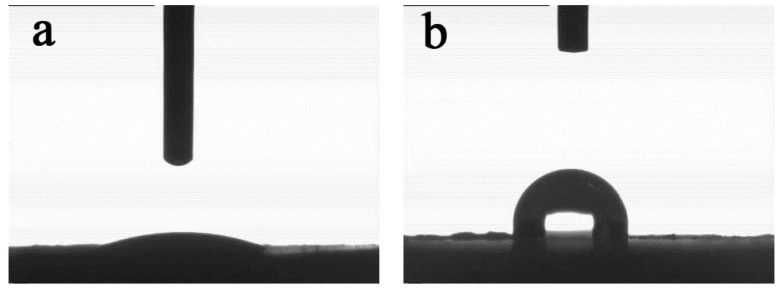
The contact angle measurement of APP (**a**) and APP@Si-171 (**b**).

As seen in Figure 5a, the average contact angle of the APP is 18°, and it can be wetting. However, the contact angle of APP@Si-171 reaches 108°, as shown in Figure 5b. All results further confirm that the hydrophobic properties of APP@Si-171 are greatly improved.

**Figure 6:**
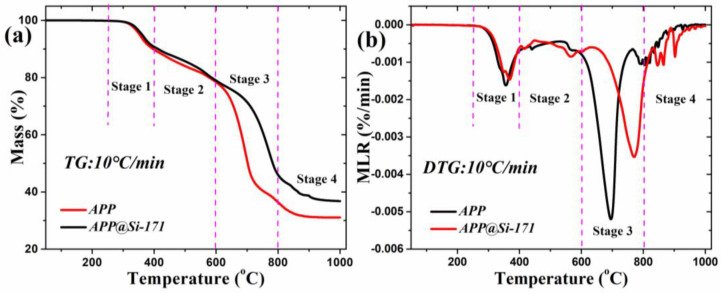
TG (**a**) and DTG (**b**) curves of APP and APP@Si-171 in N_2_ atmosphere.

To characterize the improved thermal stability of modified APP@Si-171, the measured TG and DTG curves of APP and APP@Si-171 in N_2_ atmosphere are presented and compared in Figure 6. There are four distinct stages in the thermal decomposition curves. As the temperature increases into the range of 250 to 400 °C, unstable groups such as amino groups in APP decompose and release NH_3_ as well as a small amount of H_2_O, while the mass loss of APP is about 10.2% in this temperature range. While in the second stage, about 400 to 600 °C, further temperature rise causes thermal decomposition of superphosphoric acid, which lead to the release of NH_3_ and considerable H_2_O as well as the formation of phosphoric acid, pyrophosphate, polyphosphoric acid and crosslinked P_2_O_5_ [30]. The mass loss in this stage is about 11.4%. In the third stage, about 600–850 °C, significant improvement of thermal stability is observed for APP@Si-171 based on the discrepancy of the two curves. In the APP@Si-171 curve, the magnitude of the main peak considerably declines, and the onset, peak and end temperatures are all greatly delayed. Detailed values of these parameters are listed in Table 3. The previously generated phosphoric acid, pyrophosphoric acid and polyphosphoric acid are decomposed at this stage. Finally, as the temperature exceeds 850 °C, no appreciable thermal decomposition can be detected for both materials, and the final residues for APP and APP@Si-171 are 31.1% and 36.8%, respectively. Based on these analyses, it can be concluded the added Si-171 exerts its flame resistance improvement mainly in the third stage.

The kinetics of overall thermal decomposition in the third stage, reflected by Arrhenius parameters, can also be quantitatively estimated by the corresponding equations [31,32]:

(2) $$E=\frac{eRT_{max}^{2}\frac{MLR_{max}}{m_{init}}}{\left(1-\theta \right)\frac{dT}{dt}}$$

(3) $$A=\frac{eMLR_{max}}{m_{init}}e^{\frac{E}{RT_{max}}}$$

where *E* is the activation energy, *e* is the Euler number, *A* is preexponential factor, *R* is the ideal gas constant, *T_max_* is the peak temperature, *m_init_* is the initial mass at the onset of the peak and *θ* is the ratio of mass at end temperature of peak to *m_init_*. The calculated values are also listed in Table 3. Compared with APP, the activation energy of APP@Si-171 increased from 205.9 kJ/mol to 214.9 kJ/mol, which again implies the better flame resistance after modification. Apparently, the thermal kinetic calculation results also confirm that the modified APP@Si-171 has satisfactory thermal stability.

### 3.2. Dispersion of the Fillers

**Figure 7:**
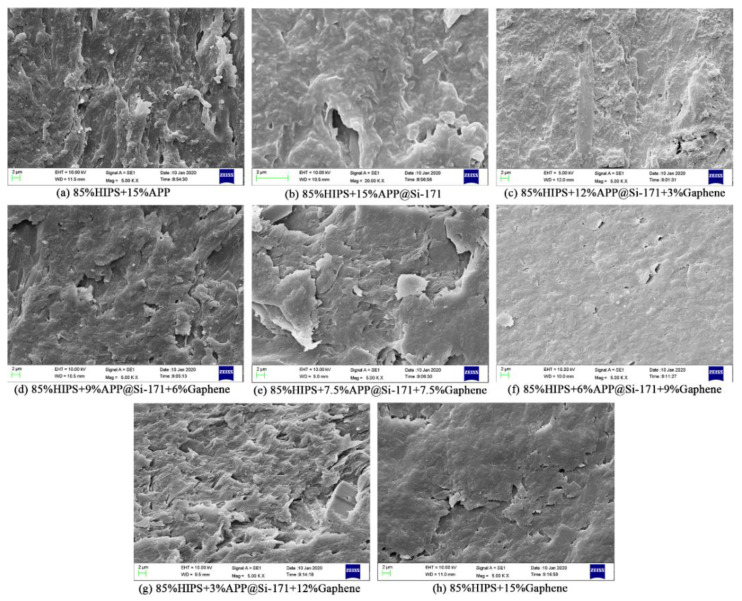
SEM images of HIPS composites (×5k): (**a**) HIPS2, (**b**) HIPS3, (**c**) HIPS4, (**d**) HIPS5, (**e**) HIPS6, (**f**) HIPS7, (**g**) HIPS8, (**h**) HIPS9.

In order to intuitively observe the dispersion of the filler in the HIPS matrix, the SEM images of the cross section of the composites are studied. As shown in Figure 7a, APP shows agglomeration and uneven dispersions in the HIPS matrix, which might affect the properties of HIPS composites. Nevertheless, after surface modification by Si-171, APP@Si-171 renders a relatively uniform dispersion in the HIPS matrix (Figure 7b), and the agglomeration is also improved. While in Figure 7c–g, where graphene is gradually added into the composites, the added multilayer graphene formed many sheet structures in the HIPS matrix, indicating good dispersion of graphene in the HIPS matrix.

### 3.3. Thermogravimetric Analysis of Improved HIPS Composites

**Figure 8:**
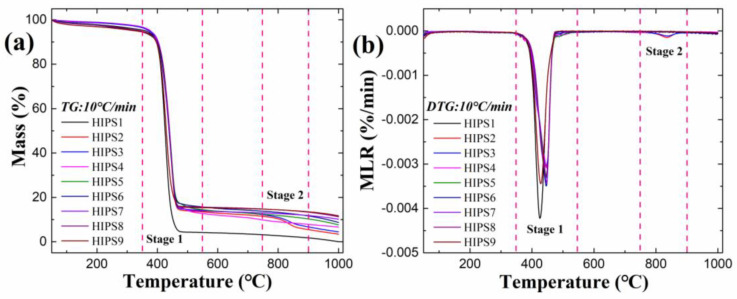
TG (**a**) and DTG (**b**) curves of HIPS composites in N_2_.

Figure 8 illustrates the TG and DTG curves of HIPS1-9 composites in the temperature of range of 50–1000 °C under 10 °C/min heating condition. A single peak is clearly identified at the temperature range of 350–550 °C in HIPS4-9 curves, corresponding to the one-step reaction of pyrolysis, whereas another minor peak is also found for HIPS2 and HIPS3 at the temperature range of 800–900 °C. According to the results in the thermal stability characterization of APP, this minor peak is associated with the thermal decomposition of APP and APP@Si-171. The pyrolysis of APP and APP@Si-171 at the main peak produces phosphoric acid, pyrophosphoric acid and polyphosphate. These productions would undergo further decomposition at 800–900 °C, which is responsible for the observed minor peak. As the graphene is added into the composite and the fraction of APP@Si-171 decreases, this minor peak vanishes.

The single peak presented in HIPS4-9 curves in Figure 8 could be attributed to the properties of the two fillers and the different controlling mechanisms in the HIPSs matrix. Czegeny et al. [33] studied the effect of APP addition on PS products and found that the presence of APP significantly reduced the yields of styrene dimer and trimer, but increased the yields of benzene, toluene, ethylbenzene, α-methylstyrene and other by-products. Due to the interaction of the side group of benzene ring with APP and polyphosphate produced by the decomposition of APP@Si-171, the electron supplying ability of the side group of benzene ring decreases, which benefits the reactivity of the second-order macromolecular free radical, the C-H bond strength of the third-order carbon atom and, accordingly, weakens the decomposition of PS. Moreover, phosphoric acid promotes hydrogen transfer, which leads to cyclization and aromatization of the aliphatic product chain, forms indene compounds and further promotes char formation. It can be seen in Figure 7 that graphene forms a large and flat graphene sheet in HIPS matrix, which helps transfer the heat released by the decomposition of polymer matrix. Meanwhile, the sheets are stacked layer by layer, forming a physical barrier that undermines heat transfer in solid and the infiltration of volatiles and smoke particles. As a result, the thermal decomposition of the HIPS4–9 samples presents a single peak.

A phenomenon that should be noticed is that the difference of TG curves at 400–800 °C is not obvious in Figure 5a. The difference is not obvious at 400–500 °C in TG curves, possibly because the pyrolysis of HIPS matrix is dominant at this stage. After 500 °C, it is mainly the pyrolysis of APP and APP@Si-171 and their products. Because the weight ratio of APP and APP@Si-171 is not much different in HIPS samples, the difference of TG curves is not obvious after 500 °C.

Utilizing the same analysis method demonstrated in Section 3.1, the related characteristic data during thermal decomposition of HIPS composites, including *T*_d, onset_, *T*_max_, MLR_max_, Residue yield, *A* and *E* are summarized in Table 4.

The data in Table 4 show that the addition of APP@Si-171 and graphene to HIPS matrix alone or jointly can both increase *T*_d, onset_ and *T*_max_. Specifically, compared with pure HIPS, the addition of 15 wt% filler would delay the *T*_d, onset_ of composite materials by 4.9 (15 wt% APP@Si-171), 17 (7.5 wt% APP@Si-171 + 7.5 wt% graphene) and 2.2 °C (15 wt% graphene). The gaseous products of NH_3_ and H_2_O during APP@Si-171 thermal decomposition could slow down the pyrolysis reaction of HIPS by diluting free radicals, and therefore, delay *T*_d, onset_. Moreover, all the *T*_max_ of the composites are delayed, which can be attributed to the fact that the added graphene advances the third stage of decomposition in HIPS3 to the main peak stage of the composite. Less APP@Si-171 in composite would directly lead to the reduction of mass loss caused by its thermal decomposition, increase *T*_max_ and decrease MLR_max_.

Compared with pure HIPS, the char residue of HIPS3 was increased from 0% to 4.4%. This is due to the promotion of char formation by APP@Si-171 and its thermal decomposition products, so that adding APP@Si-171 to HIPS3 can increase the residue yield. Moreover, the char residues of HIPS4-HIPS9 were increased gradually. As discussed above, the sheet-like structure of graphene, which blocks the volatile penetration and heat transfer in solid is credited for the increased char residue as more graphene is added. For pure HIPS, the single-step thermal decomposition with almost no residue is considered to be depolymerization and cyclization [34].

As shown in Table 4, *E* of HIPS is remarkably increased as the two fillers are added, revealing that the addition of fillers makes the decomposition of HIPS more difficult to occur, thus improving its thermal stability. Moreover, with the increase of the content of graphene, *E* increases first and then declines. The viscosity of the composite increases with the increase of graphene content, which affects the intumescence of APP and causes the decrease of *E*.

In addition, in order to study the synergistic effect of APP@Si-171 and graphene in HIPS matrix, Equations (4)–(7) are used to quantify the synergistic effect index of *T*_d, onset_, *T*_max_, residue yield (SE_RE_) and *E* of HIPS/APP@Si-171/graphene composites. The calculation results are listed in Table 5. All the calculated SE values are greater than 1. Meanwhile, the addition of fillers reduces the MLR_max_ of HIPS composite as demonstrated in Figure 8. All these results indicate that APP@Si-171 and graphene have synergistic effect on improving the thermal stability of HIPS composites.

(4) $$SE_{T_{d,onset}}=\frac{T_{d,onset}{}_{H+xAPP+yG}-T_{d,onset}{}_{H}}{\frac{x}{x+y}\left(T_{d,onset}{}_{H+\left(x+y\right)APP}-T_{d,onset}{}_{H}\right)+\frac{y}{x+y}\left(T_{d,onset}{}_{H+\left(x+y\right)G}-T_{d,onset}{}_{H}\right)}$$

(5) $$SE_{T_{max}}=\frac{T_{max}{}_{H+xAPP+yG}-T_{max}{}_{H}}{\frac{x}{x+y}\left(T_{max}{}_{H+\left(x+y\right)APP}-T_{max}{}_{H}\right)+\frac{y}{x+y}\left(T_{max}{}_{H+\left(x+y\right)G}-T_{max}{}_{H}\right)}$$

(6) $$SE_{RE}=\frac{RE_{H+xAPP+yG}-RE_{H}}{\frac{x}{x+y}\left(RE_{H+\left(x+y\right)APP}-RE_{H}\right)+\frac{y}{x+y}\left(RE_{H+\left(x+y\right)G}-RE_{H}\right)}$$

(7) $$SE_{E}=\frac{E_{H+xAPP+yG}-E_{HIPS}}{\frac{x}{x+y}\left(E_{H+\left(x+y\right)APP}-E_{H}\right)+\frac{y}{x+y}\left(E_{H+\left(x+y\right)G}-E_{H}\right)}$$

### 3.4. Cone Calorimeter Test Analysis of Improved HIPS Composites

Bench-scale cone calorimeter tests (CCT) were carried out to investigate the interaction between the two additives in the matrix and the overall thermal behaviors of relatively large samples during the combustion process. The obtained flammability properties of neat HIPS and its composites, including PHRR, THR (total heat release), CO_2_ and residue yields are listed in Table 6.

In addition, the synergistic effect of APP@Si-171 and graphene in HIPS matrix can also be assessed by Equations (8)–(10) following the similar method of Equation (1) by calculating the synergistic effect indexes of PHRR, residue yield and CO_2_ yield (SE_C_) of HIPS/APP@Si-171/graphene composites, shown in Table 7. Heat release rate is the main parameter featuring the fire hazardous of solid combustibles since it determines the received heat of unburnt fuels. The calculated SE of PHRR in Table 7 are all larger than 1, implying that the blended APP@Si-171/graphene additive exhibits good synergistic effect on improving flame retardance of HIPS composites. Although the SE of CO_2_ and residue yields in some cases are lower than 1, they are subordinate parameters in evaluating fire safety compared with that of PHRR. Furthermore, from Table 6 and Table 7, it can be easily concluded that APP@Si-171 plays a significant role in promoting CO_2_ production while graphene is significant in increasing char residue.

(8) $$SE_{PHRR}=\frac{PHRR_{H+xAPP+yG}-PHRR_{H}}{\frac{x}{x+y}\left(PHRR_{H+\left(x+y\right)APP}-PHRR_{H}\right)+\frac{y}{x+y}\left(PHRR_{H+\left(x+y\right)G}-PHRR_{H}\right)}$$

(9) $$SE_{RE}=\frac{RE_{H+xAPP+yG}-RE_{H}}{\frac{x}{x+y}\left(RE_{H+\left(x+y\right)APP}-RE_{H}\right)+\frac{y}{x+y}\left(RE_{H+\left(x+y\right)G}-RE_{H}\right)}$$

(10) $$SE_{C}=\frac{C_{H+xAPP+yG}-C_{H}}{\frac{x}{x+y}\left(C_{H+\left(x+y\right)APP}-C_{H}\right)+\frac{y}{x+y}\left(C_{H+\left(x+y\right)G}-C_{H}\right)}$$

**Figure 9:**
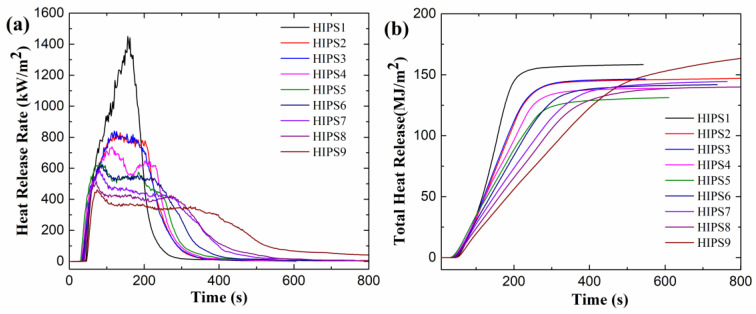
Measured HRR (**a**) and THR (**b**) curves of HIPS composites.

Figure 9 shows the HRR and THR curves of HIPS composites. Combining with Table 6, it can be found that APP@Si-171 and graphene have synergistic effect on reducing the PHRR of composites. Adding 15 wt% APP@Si-171 to HIPS matrix can reduce the PHRR and THR by about 42.0% and 7.5%, respectively. In the cone calorimeter tests, the gaseous products of NH_3_ and H_2_O generated by the thermal decomposition of APP@Si-171 dilute the gaseous small molecular products evolved by pyrolysis of HIPS. Meanwhile, the produced gases by APP@Si-171 can combine with phosphoric acid, another pyrolyzate of APP@Si-171, through foaming and promote cross-linking and char formation to form a barrier carbon layer, which hinders heat transfer and volatilization of gas products, delays the HRR rise rate of HIPS/APP@Si-171 composite and ultimately reduces its PHRR and THR. With the increase of the content of graphene, the PHRR of the composite decreases monotonously, whereas the THR declines first and then increases. This is mainly because the lamellar carbon layer formed by the interaction of graphene and APP@Si-171 blocks the heat transfer in solid and the volatilization of gaseous thermal decomposition products when the amount of graphene is small. As the amount of graphene increases, the viscosity of the condensed phase is enhanced [26], which slows down the expansion of the intumescent flame retardant APP@Si-171 and reduces the thickness of the obtained intumescent char layer and the thermal insulation performance of the intumescent layer, and therefore, the THR of the composites increases.

**Figure 10:**
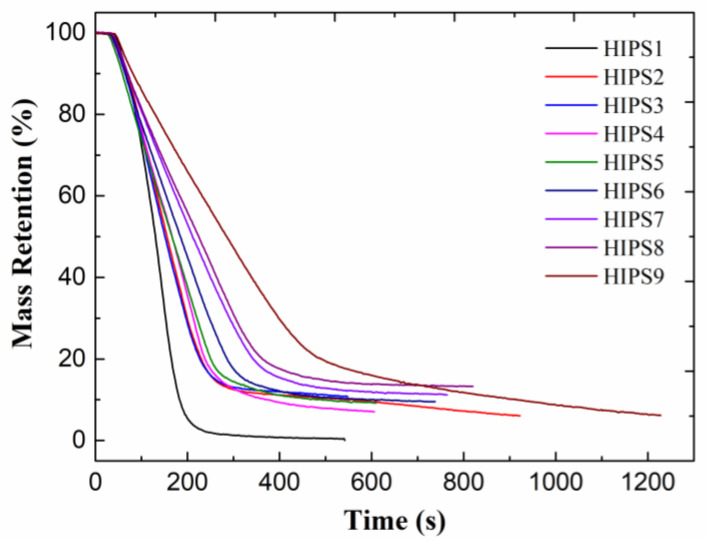
Mass retention curves of HIPS composites.

Figure 10 presents the mass retention curves of HIPS composites and it shows that adding APP@Si-171 and graphene would increase the residue yield. The addition of a small amount of graphene reduces the mass of residue of the composite; however, as the amount of graphene increases, according to Table 7, the SE_RE_ of residue yield of HIPS7 and HIPS8 are larger than 1, indicating that APP@Si-171 and graphene synergistically facilitate the generation of char residue of HIPS composites.

**Figure 11:**
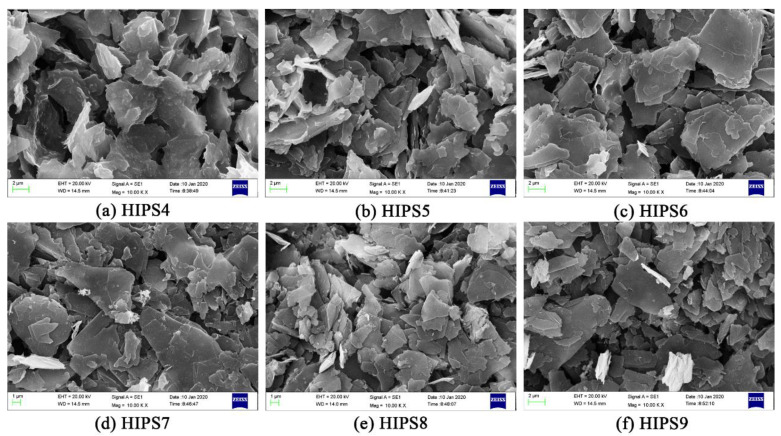
SEM images of char residues (×10k): (**a**) HIPS4, (**b**) HIPS5, (**c**) HIPS6, (**d**) HIPS7, (**e**) HIPS8, (**f**) HIPS9.

To better understand the flame retardancy mechanism, SEM experiments were also performed to investigate the microstructure of char residues of HIPS4, HIPS5, HIPS6, HIPS7, HIPS8 and HIPS9, and the results are shown in Figure 11. Sheet structure can be distinctly identified in all the subgraphs. The sheets are stacked on each other to form a dense char layer serving as a heat and volatilization barrier, which could consequently improve the flame retardancy of the composites.

## 4. Conclusions

In this work, surface-modified ammonium polyphosphate (APP@Si-171) was prepared by surface-modifying APP with Si-171, and the surface modification of APP was explored by FTIR, EDS, SEM and the static contact angle tests. FTIR and EDS results showed that APP@Si-171 was successfully modified, the SEM images of APP and APP@Si-171 showed that the microscopic particles of APP@Si-171 were well modified and the hygroscopicity and compatibility of APP were improved. The result of static contact angle tests further confirmed that the hydrophobic properties of APP@Si-171 were significantly improved. Moreover, it can also be inferred that the distribution of Si in APP@Si-171 sample was relatively uniform through EDS analysis. APP@Si-171 and graphene were individually and jointly incorporated into HIPS to prepare a series of improved composites, respectively. The results from SEM test on studying the dispersion of filler showed that APP@Si-171 was relatively uniformly dispersed and the agglomeration was also improved. Similarly, graphene was well dispersed in the HIPS matrix because the added multilayer graphene formed many sheet structures in the HIPS matrix. Subsequently, the thermal and flammability properties were explored by TGA and cone calorimeter tests. The results showed that APP@Si-171 and graphene increased the *T*_d, onset_, *T*_max_ as well as residue yield and reduced the PHRR, THR and CO_2_ yield. On the one hand, APP@Si-171 and its thermal decomposition products slowed down the thermal decomposition process and benefited the formation of char residue; on the other hand, graphene was the main component of the carbon layer, and the sheet of graphene stacked layer by layer served as a barrier to prevent the volatilization of HIPS thermal decomposition products and heat transfer. The synergistic effect between APP@Si-171 and graphene was investigated by calculating the synergistic effect index. The results showed that the synergistic effect index of *T*_d, onset_, *T*_max_, residue yield and *E* of HIPS4-HIPS8 composites were all greater than 1, and the MLR_max_ of HIPS composite was decreased by the addition of fillers, which implied that APP@Si-171 and graphene had a synergistic effect on enhancing the thermal stability and flame retardancy of HIPS. This work hopefully provides some guidance for designing or choosing a suitable flame retardant for engineering applications.

## Figures and Tables

**Table 1:** Formulations of HIPS and its composites.

Samples	HIPS (wt%)	APP (wt%)	APP@Si-171 (wt%)	Graphene (wt%)
HIPS1	100	0	0	0
HIPS2	85	15	0	0
HIPS3	85	0	15	0
HIPS4	85	0	12	3
HIPS5	85	0	9	6
HIPS6	85	0	7.5	7.5
HIPS7	85	0	6	9
HIPS8	85	0	3	12
HIPS9	85	0	0	15

**Table 2:** Test analysis of the electron energy spectrum weight and atomic percentage of APP@Si-171.

Element Line	Weight (%)	Atom (%)
O K	45.95	47.52
Si K	0.15	0.09
C K	15.59	21.47
Al K	0.46	0.28
P K	21.75	11.62
N K	16.10	19.01

**Table 3:** Detailed information of the main peaks in the DTG curves of both flame retardants.

	*T*_d, onset_ (°C)	*T*_max_ (°C)	*T*_end_ (°C)	MLR_max_ (‱/min)	Mass Residues (%)	*A* (s^−1^)	*E* (kJ/mol)
**APP**	317.2	694.3	915.2	0.52	31.1	11.2	205.9
**APP@Si-171**	330.3	768.7	952.3	0.35	36.8	9.1	214.9
**Discrepancy**	13.1	74.4	37.1	0.17	5.7	2.1	9.0

**Table 4:** Thermal decomposition parameters of HIPS composites.

Samples	*T*_d, onset_ (°C)	*T*_max_ (°C)	MLR_max_ (‱/min)	Residue Yield (%)	*A* (s^−1^)	*E* (kJ/mol)
HIPS1	404.8	424.7	0.42	0.0	10.2	71.66
HIPS2	410.3	447.3	0.31	3.4	9.6	74.0
HIPS3	409.7	446.8	0.30	4.4	9.6	75.27
HIPS4	415.2	448.3	0.31	6.6	9.2	78.2
HIPS5	415.8	447.2	0.33	7.6	9.3	81.4
HIPS6	421.8	446.3	0.35	8.7	9.6	86.3
HIPS7	416.2	447.3	0.34	10.1	9.6	82.2
HIPS8	417.5	444.8	0.33	11.2	9.4	81.2
HIPS9	407.0	427.8	0.34	11.7	9.1	75.3

**Table 5:** Synergistic effect of APP@Si-171 and graphene on HIPS composites.

Samples	SE of *T*_d, onset_	SE of *T*_max_	SE of Residue Yield	SE of *E*
HIPS4	2.40	1.28	1.12	1.78
HIPS5	2.92	1.55	1.03	2.65
HIPS6	4.85	1.71	1.08	3.97
HIPS7	3.50	2.10	1.16	2.86
HIPS8	4.69	2.89	1.10	2.58

**Table 6:** Measured flammability parameters of HIPS and its composites in cone calorimeter tests.

Samples	THR (MJ/m^2^)	PHRR (kW/m^2^)	CO_2_ Yield (kg/kg)	Residue Yield (%)
HIPS1	158.3	1450.1	5.5	0
HIPS2	147.6	830.0	7.1	6.1
HIPS3	146.5	840.7	4.3	10.8
HIPS4	138.8	739.3	3.7	7.1
HIPS5	131.2	635.7	3.5	9.2
HIPS6	141.8	625.0	4.2	9.6
HIPS7	144.5	588.3	3.6	11.2
HIPS8	140.0	516.2	3.8	13.3
HIPS9	177.2	461.6	3.7	6.1

**Table 7:** Calculated synergistic indexes of PHRR, CO_2_ yield and residue yields.

Samples	SE of PHRR	SE of CO_2_ Yield	SE of Residue Yield
HIPS4	1.04	1.01	0.72
HIPS5	1.07	1.12	0.93
HIPS6	1.03	0.75	0.97
HIPS7	1.03	1.16	1.13
HIPS8	1.02	0.94	1.34

## Data Availability

The data presented in this study are available on request from the corresponding author.
